# Epigallocatechin-3-Gallate (EGCG) Suppresses Pancreatic Cancer Cell Growth, Invasion, and Migration partly through the Inhibition of Akt Pathway and Epithelial–Mesenchymal Transition: Enhanced Efficacy When Combined with Gemcitabine

**DOI:** 10.3390/nu11081856

**Published:** 2019-08-09

**Authors:** Ran Wei, Natalia E. Cortez Penso, Robert M. Hackman, Yuefei Wang, Gerardo G. Mackenzie

**Affiliations:** 1 Tea Science Institute, Zhejiang University, Hangzhou 310058, China; 2 Department of Nutrition, University of California, Davis, CA 95616, USA

**Keywords:** pancreatic cancer, epigallocatechin-3-gallate, Akt, EMT, gemcitabine

## Abstract

Most pancreatic cancers are usually diagnosed at an advanced stage when they have already metastasized. Epigallocatechin-3-gallate (EGCG), a major polyphenolic constituent of green tea, has been shown to reduce pancreatic cancer growth, but its effect on metastasis remains elusive. This study evaluated the capacity of EGCG to inhibit pancreatic cancer cell migration and invasion and the underlying mechanisms. EGCG reduced pancreatic cancer cell growth, migration, and invasion in vitro and in vivo. EGCG prevented “Cadherin switch” and decreased the expression level of TCF8/ZEB1, β-Catenin, and Vimentin. Mechanistically, EGCG inhibited the Akt pathway in a time-dependent manner, by suppressing IGFR phosphorylation and inducing Akt degradation. Co-treatment with catalase or N-Acetyl-L-cysteine did not abrogate EGCG’s effect on the Akt pathway or cell growth. Moreover, EGCG synergized with gemcitabine to suppress pancreatic cancer cell growth, migration, and invasion, through modulating epithelial–mesenchymal transition markers and inhibiting Akt pathway. In summary, EGCG may prove beneficial to improve gemcitabine sensitivity in inhibiting pancreatic cancer cell migration and invasion, to some extent through the inhibition of Akt pathway and epithelial–mesenchymal transition.

## 1. Introduction

Pancreatic cancer is a worldwide problem, being one of the most deadly malignancies [1]. Due to the lack of detection and prognosis methods, patients with pancreatic cancer are usually diagnosed at an advanced stage when the cancer has already metastasized [2]. Gemcitabine, a deoxycytidine analogue commonly used as a first-line therapy for metastasis pancreatic cancer patients, extends pancreatic cancer patients’ life by only months. Thus, the identification of strategies that can prevent or target metastatic disease is of critical importance to improve the patient’s outcome [3].

Akt activation is frequent in pancreatic cancer [4,5,6], playing a critical role in promoting tumor growth [7]. In addition, Akt activation is frequently found in metastatic cancer, being considered as a “master regulator” of cell metastasis [8]. Akt is responsible for transducing extracellular signal, like insulin, insulin growth factors (IGF), and epithelial growth factors (EGF) to initiate intracellular signaling cascade, such as mTOR and GSK3β. Activation of Akt is indicated in the epithelial–mesenchymal transition (EMT), in control of cell–cell junctions, cell polarization, motility, and cell-matrix adhesion [9]. Of note, emerging evidence has indicated that Akt activation and EMT are closely associated with drug resistance characteristics of pancreatic cancer cells [7,10]. Thus, the Akt pathway is recognized as a potential drug target for pancreatic cancer metastasis.

Epigallocatechin-3-gallate (EGCG), a major polyphenolic constituent of green tea, possesses preventive and therapeutic properties in various cancer models via different mechanisms, including pro-oxidant, anti-inflammation, modulating cell cycle, and apoptosis, etc. [11,12]. The number and the specific position of its hydroxyl make EGCG more active than other tea catechins in its anti-cancer effect [13,14]. The evidence for a beneficial effect of EGCG on cancer metastasis is more sparse [15,16], with the exact cellular targets related to cancer metastasis not being completely elucidated.

In the present study, we investigated the effect and mechanism of action of EGCG alone and in combination with gemcitabine on pancreatic cancer cell growth, migration, and invasion. Our results show that EGCG alone or combined with gemcitabine strongly reduced pancreatic cancer cell growth, migration, and invasion by inhibiting the Akt pathway and modulating EMT markers.

## 2. Materials and Methods

### 2.1. Chemicals and Reagents

EGCG (≥98% purity) was purchased from Tocris (Minneapolis, MN). 3-(4,5-dimethylthiazol-2-yl)-2,5-diphenyltetrazolium bromide (MTT) (≥97.5%), Catalase, N-Acetyl-L-cysteine, RIPA lysis buffer, Halt Protease Inhibitor Cocktail, and Phosphatase Inhibitor Cocktail were purchased from MilliporeSigma (St. Louis, MO, USA). Gemcitabine-HCL (>99%) was purchased from BIOTANG (Waltham, MA, USA). Transwell cell culture inserts (8.0 µm) and multiwell 24-well support plates were purchased from Corning (Corning, NY, USA). TRIzol^®^ reagent, High-Capacity cDNA reverse transcription kits, and SuperSignal™ West Dura Extended Duration Substrate were purchased from ThermoFisher Scientific (Waltham, MA, USA). Direct-zol RNA MiniPrep kits were purchased from ZYMO Research (Irvine, CA, USA). Primers for qPCR were purchased from Integrated DNA Technologies (Coralville, IA, USA). Antibodies for Western blot were purchased from Cell Signaling Technology (Danvers, MA, USA). Bradford protein assay reagent, 30% (*w*/*v*) Acrylamide/Bis Solution, 4 × Laemmli sample buffer, Immun-Blot^®^ PVDF Membranes, and SsoAdvanced^TM^ SYBR^®^ Green supermix were purchased from Bio-Rad (Hercules, CA, USA). Protein A/G PLUS-Agarose was purchased from Santa Cruz Biotechnology (Dallas, TX, USA). Akt1 untagged clone was purchased from OriGene (Rockville, MD, USA). The Akt inhibitor MK2206 was purchased from Selleckchem (Houston, TX, USA).

### 2.2. Cell Culture

The human pancreatic cancer cell lines (Panc-1, MIA PaCa-2, BxPC-3, HPAF-II, CFPAC-1, and Su.86.86,) as well as the human pancreatic normal epithelial (HPNE) cells were purchased from the American Type Culture Collection (Manassas, VA, USA). The murine FC1245 pancreatic cancer cells (KPC cells) were a gift from Dr. David Tuveson (Cold Spring Harbor Laboratory, Cold Spring Harbor, NY, USA). All cell lines were cultured in the specific medium in the conditions specified by the vendor.

### 2.3. Cell Viability

Following the treatment with various concentrations of EGCG alone or in combination with gemcitabine for 24 h and 48 h, the reduction of 3-(4,5-dimethylthiazol-2-yl)-2,5-diphenyltetrazolium bromide dye (MTT) was determined following the manufacture’s protocol (MilliporeSigma, St. Louis, MO, USA).

### 2.4. Wound Healing Assay

MIA PaCa-2 and Panc-1 cells were cultured in 6-well plates overnight. The next day, a straight scratch was made in the center of each well using a micropipette tip. Cells were then washed with PBS and treated with EGCG, gemcitabine, or both for 48 h. Cell motility was assessed by measuring the movement of the cells into the scratch area after 48 h of treatment with the various drugs.

### 2.5. Cell Migration and Invasion

For the migration assay, MIA PaCa-2 and Panc-1 cells (5.0 × 10^4^ cells per well) were plated in the top chamber inserts with polyester membrane (8 μm pore size) of 24-well plates (Corning, New York, NY, USA) overnight. The bottom chambers of the plates were filled with DMEM containing 10% (*v*/*v*) FBS. The various drug treatments, suspended in serum-free DMEM, were added to the upper chamber, and 0.75 mL 10% (*v*/*v*) FBS medium was added into the lower compartment. After 24 h of treatment, cells in the top chamber were removed using a cotton swab, and cells that migrated to the bottom chambers were fixed in methanol and stained with 0.1% (*w*/*v*) crystal violet for 0.5 h at room temperature. The stained cells were counted in 3 independent fields under an AE2000 inverted microscope (Motic, Carlsbad, CA, USA). For the invasion assay, a similar protocol was followed apart from the replacement of the top chamber of the transwell plate coated with 50 μL Matrigel (Corning, New York, NY, USA).

### 2.6. Western Blot

Following treatment with drugs, cells were lysed and whole cell fractions were isolated as previously described [17]. Proteins (10–30 μg) were separated by 10%–12% (*w*/*v*) polyacrylamide gel electrophoresis and electroblotted to PVDF membranes. After blocking with non-fat milk for 1 h, membranes were incubated overnight with the following primary antibodies (1:1000 dilution) from Cell Signaling Technology (Danvers, MA, USA): E-Cadherin (Cat #3195), N-Cadherin (Cat #13116), TCF8/ZEB1 (Cat #3396), β-Catenin (Cat #8480), Vimentin (Cat #5741), phospho-Akt (Ser473) (Cat #4060), Akt (Cat #9272), phospho-IGF-I Receptor β (Tyr1135/1136) (Cat #3024), IGF-I Receptor β (Cat #9750), phospho-PI3 Kinase p85 (Tyr458)/p55 (Tyr199) (Cat 4228), PI3 Kinase p85 (Cat 4257), phospho-mTOR (Ser2448) (Cat #5536), mTOR (Cat #2983). β-Actin (Cat #8457) was used at the same time as a loading control. After incubation with the secondary antibody (HRP-conjugated; 1:5000 dilution) for 1 h, at room temperature, the conjugates were developed and visualized using a Molecular Imager FX^TM^ System (BioRad; Hercules, CA, USA).

### 2.7. Cycloheximide Chasing Assay

Following treatment with EGCG for 6 h, cells were incubated with cycloheximide (CHX) for an additional 1–3 h. Cells were then collected, and total cell lysates were obtained for Western blot analysis.

### 2.8. Immunoprecipitation and Ubiquitination Analysis

Following treatment with EGCG for 12 h, cells lysates were prepared as described previously [18,19]. First, 500 µg of total cell proteins were pre-cleared for 30 min using 0.25 µg of the appropriate control IgG from the host species together with 20 µL of Protein A/G-Agarose. The pre-cleared-supernatant fractions were immunoprecipitated using an anti Akt antibody (1 µg), or a control IgG, together with 25 µL Protein A/G-Agarose overnight at 4 °C. After centrifugation, the beads were washed two times with PBS, and the final pellets were resuspended in 30 µL of 1 × sample buffer. After boiling, samples (15 µL of each) were immediately used for immunoblotting with anti-Ubiquitin and anti-Akt antibodies. Of note, there was no evidence of non-specific immunoprecipitation when control samples were immunoprecipitated with control IgG instead of the Akt antibody.

### 2.9. Quantitative Real-Time Polymerase Chain Reaction (qRT-PCR)

Total RNA was extracted and purified by using the TRIzol^®^ reagent and a Direct-zol RNA MiniPrep kit. High-Capacity cDNA Reverse Transcription kits were used to synthesize and amplify single-stranded cDNA according to the manufacturer’s instructions (ThermoFisher Scientific, Waltham, MA, USA). Quantitative real-time PCR was performed with a CFX96 Real-Time System (BioRad, Hercules, CA, USA) using an SsoAdvanced^TM^ SYBR^®^ Green supermix. The specific primers used for Akt were: forward (5′-3′): CACACCACCTGACCAAGATG, and reverse (5′-3′): CCTCAGAGACACGGCCTTAG [20]. The relative mRNA expression level of Akt was calculated by the 2^−∆∆CT^ method and β-Actin was used as a control. The sequence of β-Actin was: forward (5′-3′): AGAAAATCTGGCACCACACC, and reverse (5′-3′): AGAGGGTACAGGGATAGCA [21].

### 2.10. Gene Overexpression

For gene overexpression, cells were plated in 6-well plates, cultured overnight, and transiently transfected with Akt1 cDNA for 8 h. Lipofectamine^TM^ 3000 reagent was used for transfection based on the manufacturer’s instructions (ThermoFisher Scientific, Waltham, MA, USA). Following transfection, cells were treated with EGCG for 72 h and cell viability was tested. The gene overexpression efficiency was determined by immunoblotting.

### 2.11. Clonogenic Assay

Panc-1 and MIA PaCa-2 cells, plated in 60 mm plates, were treated with the test drugs for 48 h. Following treatment, cells were trypsinized, resuspended in fresh medium, counted by a Countess II FL Automated Cell Counter (ThermoFisher Scientific, Waltham, MA, USA), re-seeded (1000 cells per plate), and incubated for another 18 days. Media was replaced once weekly during the incubation. On the last day, colonies were fixed by methanol and stained with 0.1% (*w*/*v*) crystal violet in phosphate buffer saline (pH 7.4) [19]. Cells were then rinsed with distilled water, air-dried, and colonies were counted and analyzed using ImageJ software.

### 2.12. Animal Study

The animal study was approved by the University of California, Davis Animal Care and Use Committee. C57BL/6J mice (4–6 weeks) were injected subcutaneously with 0.3 × 10^6^ KPC cells per tumor suspended in 100 µL of sterile PBS. When the cells reached palpable tumor size (~300 mm^3^), mice (n = 5/group) were randomly divided among 3 groups: Control, EGCG 10 mg/kg, or EGCG 20 mg/kg. The EGCG was suspended in PBS and given once daily by *i*.*p*. injection for 10 days. Control mice were injected only with PBS. The doses of EGCG were chosen based on previous published reports [22]. Tumor size and body weight were measured every two days, and tumor size was determined using the following formula: Length × width × (length + width/2) × 0.56, in millimeters [18]. At the conclusion of the intervention, mice were euthanized, serum collected for biochemical assay determinations, and tumor weights measured. Tumor tissue was stored for future analysis.

### 2.13. Immunohistochemistry

Immunohistochemistry for E-Cadherin and N-Cadherin was conducted as previously described [23,24]. Briefly, paraffin-embedded sections (5 μm thick) were deparaffinized and rehydrated, followed by antigen retrieval performed by microwave-heating in 0.01 M citrate buffer (pH 6.0) for 10 min. H_2_O_2_ (3%) was then applied to block endogenous peroxidase activity. Following blocking for 60 min with serum, slides were incubated with primary antibody (E-Cadherin, Cat #3195; N-Cadherin, Cat #13116; Cell Signaling Technology, Danvers, MA, USA) or control IgG (dilution 1/200) overnight at 4 °C. The next day, slides were washed thrice with PBS for 5 min each time. The biotinylated secondary antibody and the streptavidin-biotin complex (Invitrogen, Carlsbad, CA, USA) were added, each for 1 h at room temperature with an interval washing. After the last wash with PBS, slides were stained with 3,3′-Diaminobenzidine tetrahydrochloride hydrate (DAB) solution, then rinsed with distilled water, counterstained with hematoxylin. Slides were then dehydrated overnight, coverslipped, and images were taken at 100 × magnification. At least 5 fields per sample were analyzed and scored using Image J software based on the intensity of each field.

### 2.14. Statistical Analysis

Data and immunoblots were obtained from at least three independent biological experiments and the obtained results were expressed as mean ± SD. Data were analyzed by one-factor analysis of variance (ANOVA) followed by the Duncan test for multiple comparisons. *t*-tests were used to analyze the difference between two groups. *p* < 0.05 was regarded as statistically significant.

## 3. Results

### 3.1. EGCG Reduces Pancreatic Cancer Cell Growth In Vitro and In Vivo

**Figure 1:**
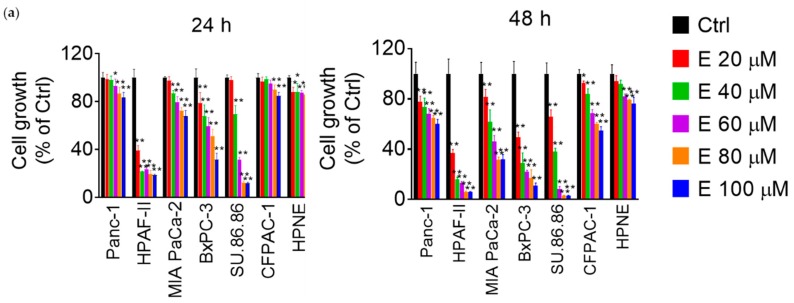

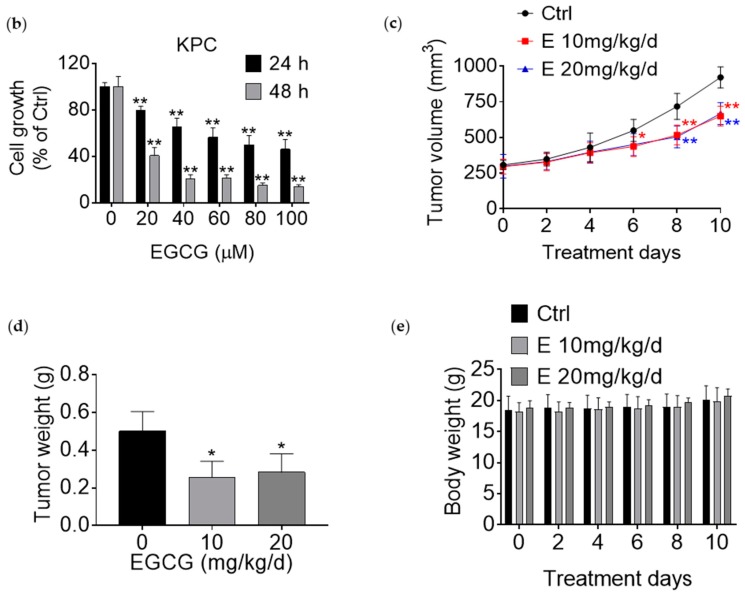
Epigallocatechin-3-gallate (EGCG) reduces pancreatic cancer cell growth in vitro and in vivo. (**a**) EGCG inhibits human pancreatic cancer cell growth in a concentration- and time-dependent manner. Cell growth was determined in Panc-1, MIA PaCa-2, HPAF-II, BxPC-3, SU- 86.86, CFPAC-1, and KPC pancreatic cancer cells, and in the human pancreatic normal epithelial (HPNE) cells after treatment with increasing EGCG concentrations for 24 or 48 h. Results are expressed as a percentage of control. * *p* < 0.05, ** *p* < 0.01 vs. control. (**b**) EGCG inhibits mouse pancreatic cancer cell KPC growth. Results are expressed as a percentage of control. ** *p* < 0.01 vs. control. (**c**) EGCG reduces xenograft tumor growth. KPC tumor volume over time of control (Ctrl), EGCG 10 mg/kg/d (■), and EGCG 20 mg/kg/d (▲) treated mice. Results are presented as the mean ± SD. * *p* < 0.05, ** *p* < 0.01 vs. control. (**d**) Tumor weight at the end of the study for control and EGCG treated groups. Results are presented as the mean ± SD. * *p* < 0.05, vs. control. (**e**) Mice body weight during treatment days for control and EGCG treated groups. Results are presented as the mean ± SD.

We first evaluated the effect of EGCG on pancreatic cancer cell growth and compared it to that of human pancreatic normal epithelial cells (HPNE). For this purpose, we treated six human pancreatic cancer cells as well as the HPNE cells with increasing concentrations of EGCG (20–100 μM) for 24 h and 48 h. As shown in Figure 1a, pancreatic cancer cell lines presented different degrees of sensitivity to EGCG. For example, the HPAF-II and SU.86.86 cells were sensitive to EGCG, with EGCG at 40 µM for 48 h reducing cell growth by 84% and 62% of control, respectively. In contrast, EGCG at 40 µM for 48 h reduced the growth of Panc-1 and CFPAC-1 cells by 27% and 17% compared to control, respectively. MIA PaCa-2 cells were moderately sensitive to EGCG, with growth being reduced by 38% under the same experimental conditions. Interestingly, EGCG had minimal effects on HPNE cell growth, and after treatment with EGCG at 40 µM for 48 h, cell growth was only reduced by 9% compared to controls (Figure 1a). Given their moderate and low sensitivity to EGCG, as well as their differential effect to chemotherapeutics [25], we chose MIA PaCa-2 and Panc-1 cells for the subsequent studies.

We next explored the chemotherapeutic potential of EGCG in murine pancreatic cancer models. First, as shown in Figure 1b, EGCG inhibited the growth of mouse pancreatic cancer KPC cells in culture in a time- and concentration-dependent manner. Next, KPC cells were injected subcutaneously into immunocompetent mice, which led to exponentially growing tumors. When the size of the tumors was ~300 mm^3^, the mice were treated either with EGCG at 10 mg/kg, EGCG at 20 mg/kg, or with PBS (vehicle control). At day 10, the tumor volumes (mean ± SD) for the vehicle control, EGCG 10 m/kg, and EGCG 20 mg/kg groups were 921.5 ± 74.7 mm^3^, 650.2 ± 69.3 mm^3^, and 668.2 ± 76.9 mm^3^, respectively (*p* < 0.01 for both; Figure 1c). At sacrifice, EGCG 10 mg/kg and EGCG 20 mg/kg reduced tumor weight by 49% and 45%, respectively, compared to vehicle-treated controls (*p* < 0.05 for both; Figure 1d).

Of note, EGCG, at both doses, was well tolerated during the experimental period. The EGCG-treated mice displayed no weight loss or other signs of toxicity (Figure 1e). For example, on the last day of the experimental period, the mean body weights in the three groups were as follows: Control = 20.1 ± 2.2 g, EGCG 10 mg/kg = 19.8 ± 2.2 g, and EGCG 20mg/kg = 20.7 ± 1.1 g. Moreover, no adverse effects of EGCG on liver and kidney function were noted, with no changes in the levels of multiple liver enzymes and kidney markers among the groups (Table 1).

### 3.2. EGCG Inhibits Pancreatic Cancer Cell Migration and Invasion by Modulating the Epithelial–Mesenchymal Transition (EMT)

**Figure 2:**
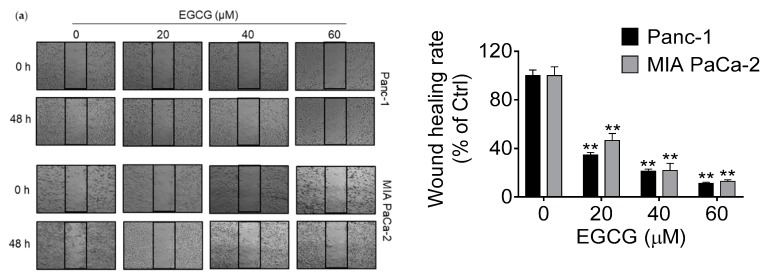

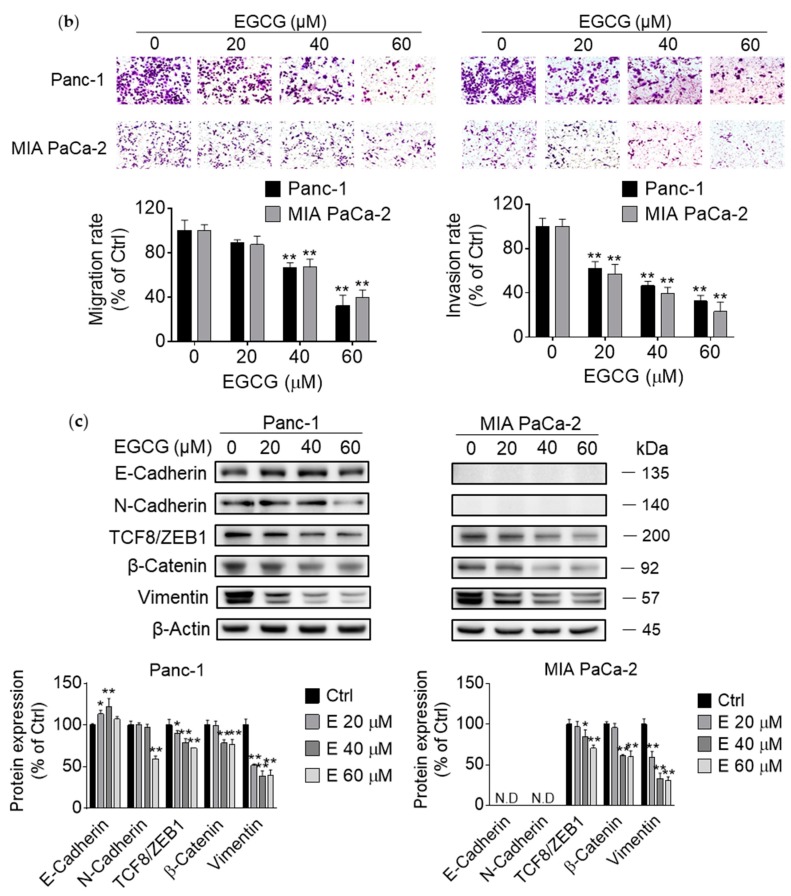

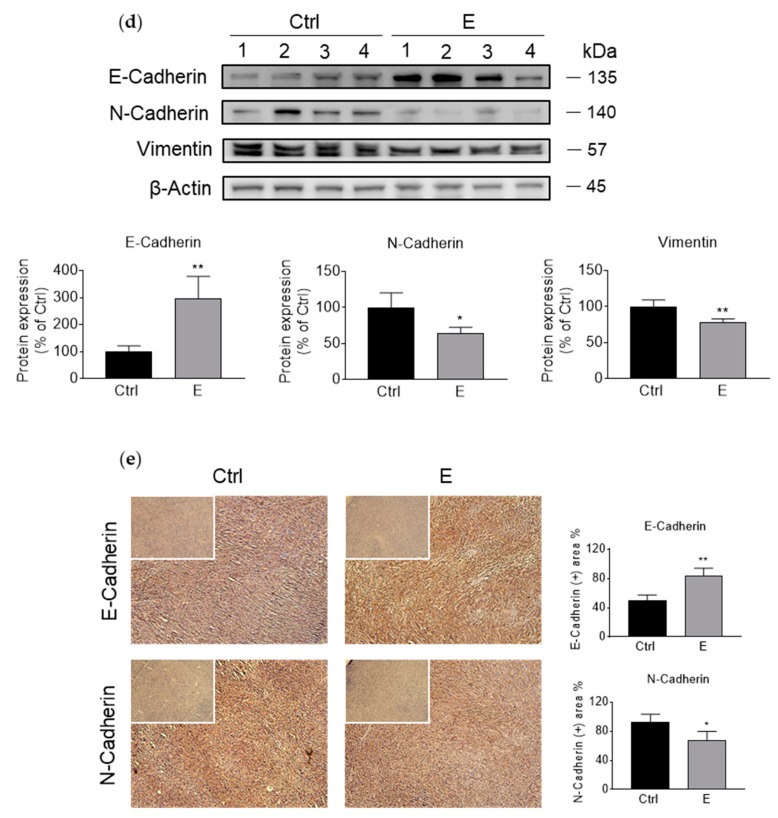
EGCG inhibits cell motility, migration and invasion in pancreatic cancer cells. (**a**) Wound healing assays were performed in Panc-1 and MIA PaCa-2 cells treated without (control) or with EGCG for 48 h. Images were visualized at time 0 h and 48 h. Quantitative analysis was conducted as described under Materials and Methods. Results are expressed as the percentage of control. ** *p* < 0.01 vs. control. (**b**) Effect of EGCG on cell migration and invasion was determined by using the inserted cartridge with or without matrigel. Briefly, Panc-1 and MIA PaCa-2 cells were treated without (control) or with EGCG for 48 h. Cells were seeded in the upper compartment of a migration or invasion chamber in serum-free culture media. After 48 h, cells that remained in the upper chamber were removed and cells that had migrated (left) or invaded (right) onto the lower surface of the membrane were fixed and stained with crystal violet. Representative images are shown (×200). The relative-fold migration and invasion values of EGCG-treated cells were normalized against the vehicle-treated cells (control) and expressed as percentage of control, which was assumed to be 100 percent. ** *p* < 0.01 vs. control. (**c**) EGCG inhibits cell migration and invasion through modulating EMT in pancreatic cancer cells. Immunoblots for E- Cadherin, N-Cadherin, TCF8/ZEB1, β-Catenin, and Vimentin in total cell protein extracts from Panc-1 and MIA PaCa-2 cells treated with escalating concentrations of EGCG, as indicated, for 48 h. Loading control: β-Actin. Bands were quantified and results are expressed as a percentage of control. * *p* < 0.05, ** *p* < 0.01 vs. control. (**d**) EGCG affects epithelial–mesenchymal transition (EMT) protein expression in vivo. Immunoblots for E-Cadherin, N-Cadherin, and Vimentin of tumor samples obtained from EGCG-treated mice. β-Actin was evaluated as the loading control. Each lane represents a different tumor sample. Bands were quantified and results are expressed as a percentage of control. * *p* < 0.05, ** *p* < 0.01 vs. control. (**e**) Tumor tissue sections were immunohistochemically stained with E-cadherin and N-cadherin. The consecutive section was stained with isotype IgG as negative staining control and shown in the upper left corner of each image. Results were quantified based on the intensity of each field and expressed as percent of E/N-Cadherin positive area per field. Images were taken at ×100. * *p* < 0.05, ** *p* < 0.01 vs. control.

We examined the capacity of EGCG to regulate cell migration and invasion. First, we performed a wound healing assay to evaluate the effect of EGCG on cell motility. After 48 h of treatment, EGCG significantly inhibited wound healing capacity in a concentration-dependent manner in both cell lines tested (Figure 2a). Compared to vehicle-treated controls, EGCG at 40 μM reduced cell motility by 79% and 78% in Panc-1 and MIA PaCa-2 cells, respectively (*p* < 0.01 for both). This pattern was consistent with the effect of EGCG on cell migration and invasion (Figure 2b). For example, EGCG at 40 μM reduced cell migration in Panc-1 and MIA PaCa-2 cells by 34% and 33% (*p* < 0.01 for both; Figure 2b). A somewhat stronger inhibitory effect by EGCG was observed determining the invasion rate. EGCG reduced cell invasion in Panc-1 and MIA PaCa-2 cells by 54% and 60%, respectively (*p* < 0.01 for both; Figure 2b). Mechanistically, EGCG reduced select markers involved in the EMT process. After 48 h of treatment, EGCG increased the expression of E-Cadherin, while it reduced the levels of N-Cadherin in Panc-1, and decreased the expression levels of multiple mesenchymal markers, including TCF8/ZEB1, β-Catenin, and Vimentin in both cell lines (Figure 2c). Consistent findings were obtained in pancreatic tumor xenografts. Compared with control group, the expression of E-Cadherin significantly increased (*p* < 0.01), whereas levels of N-Cadherin and Vimentin were reduced in the 10 mg/kg/d EGCG treated group (*p* < 0.05 and *p* < 0.01; Figure 2d,e), as evaluated by Western blot and Immunohistochemistry. These results suggest a functional role of EGCG in inhibiting pancreatic cancer cell invasion and migration by regulating EMT markers.

### 3.3. EGCG Inhibits Akt Signaling Pathway In Vitro and In Vivo

**Figure 3:**
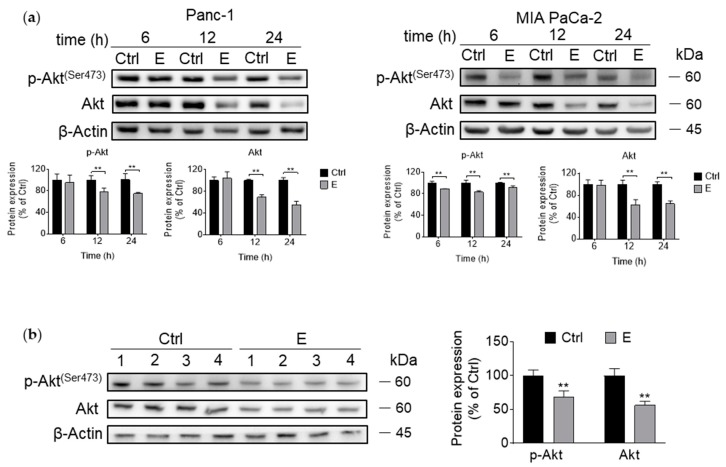

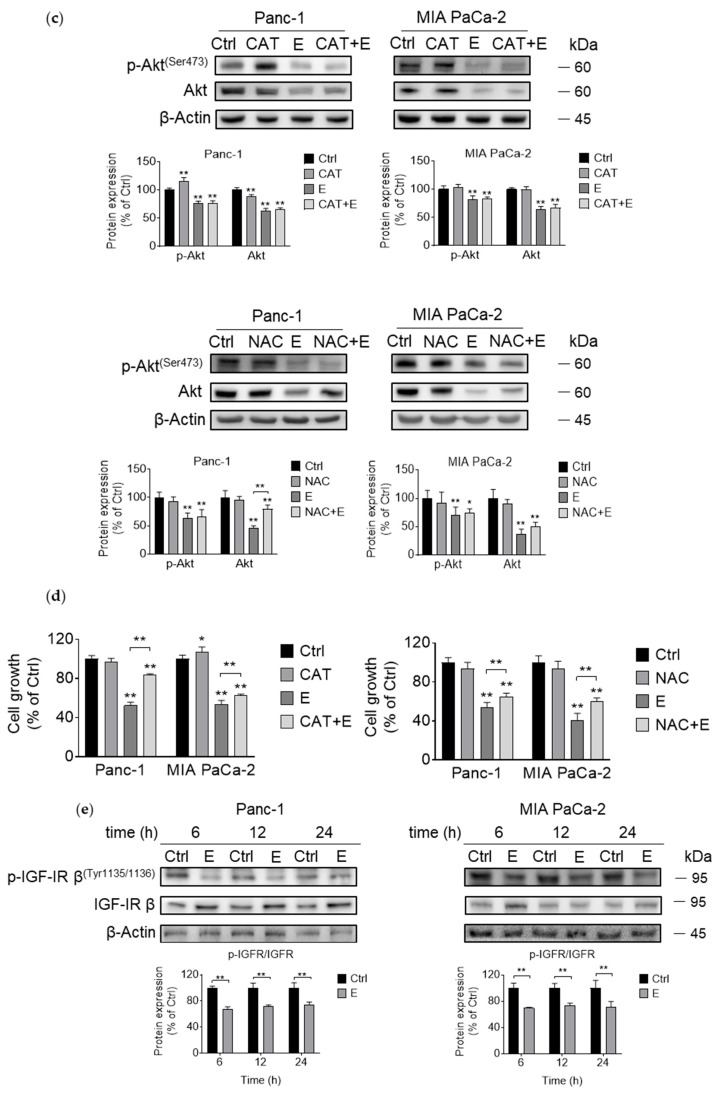

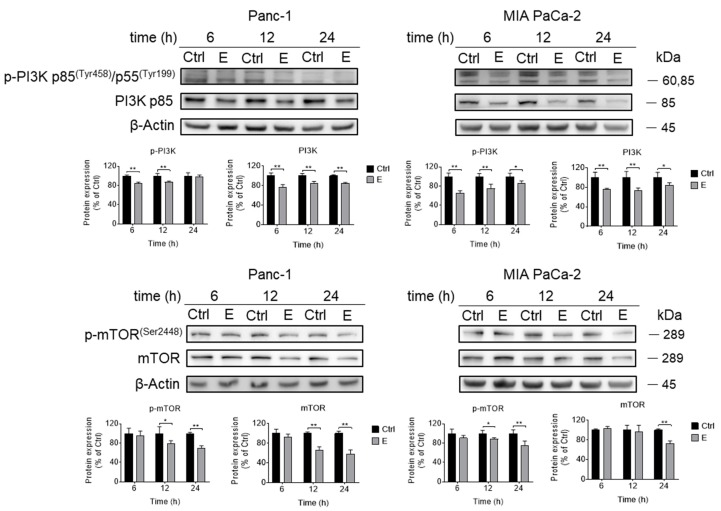
EGCG inhibits Akt signaling pathway in pancreatic cancer cells. (**a**) EGCG inhibited both phosphorylated and total Akt expression in vitro. Immunoblots for phosphorylated and total Akt in total cell protein extracts from Panc-1 and MIA PaCa-2 cells treated with EGCG for various time points. Loading control: β-Actin. Bands were quantified and results are expressed as a percentage of control. ** *p* < 0.01 vs. control. (**b**) Both phosphorylated and total Akt expression levels were reduced in tumor samples obtained from EGCG-treated mice. β-Actin was evaluated as the loading control. Each lane represents a different tumor sample. Bands were quantified and results are expressed as a percentage of control. ** *p* < 0.01 vs. control. (**c**) Catalase (CAT) and N-Acetyl-L-cysteine (NAC) failed to prevent the effect of EGCG (E) on the expression of the Akt pathway. Immunoblots for phosphorylated and total Akt in total cell protein extracts from Panc-1 and MIA PaCa-2 cells treated with EGCG (E), without or with catalase (CAT) or N-Acetyl-L-cysteine (NAC) for 12 h. Loading control: β-Actin. Bands were quantified and results are expressed as a percentage of control. * *p* < 0.05, ** *p* < 0.01 vs. control. (**d**) Catalase (CAT) and N-Acetyl-L-cysteine (NAC) partly ameliorate the cell growth inhibitory effect induced by EGCG (E). Results are expressed as a percentage of control. * *p* < 0.05, ** *p* < 0.01 vs. control. (**e**) EGCG inhibits upstream and downstream events in the Akt pathway in pancreatic cancer cells. Expression of phosphorylated/total IGFR, phosphorylated and total PI3K, and phosphorylated and total mTOR was tested at the same condition. Loading control: β-Actin. Bands were quantified and results are expressed as percentage of control. * *p* < 0.05, ** *p* < 0.01 vs. control.

We examined whether EGCG affected the cell metastasis through Akt pathway in pancreatic cancer. First, we examined the effect of EGCG on Akt activation at various time points by measuring the levels of Akt phosphorylation. As shown in Figure 3a, EGCG decreased the levels of both phosphorylation and total Akt in both cell lines in a time-dependent manner. Consistent with the in vitro results, EGCG reduced the levels of p-Akt and total Akt (*p* < 0.01) in pancreatic tumor xenograft samples (Figure 3b).

An important consideration when exploring EGCG’s mechanism of action is that in the presence of oxygen, EGCG undergoes auto-oxidation to generate reactive oxygen species (ROS) and affect cell growth by inducing cell death [26]. Therefore, to elucidate whether the effect of EGCG on Akt is mediated by the increase in ROS, Panc-1 and MIA PaCa-2 cells were incubated with or without EGCG at 40 µM in the presence or absence of CAT or NAC for 12 h. Co-treatment with CAT or NAC reversed, in part, the effect of EGCG on Akt inhibition (Figure 3c).

We then examined whether catalase and NAC could prevent the reduction in pancreatic cancer cell growth induced by EGCG. After 72 h of treatment, EGCG at 40 µM reduced cell growth by 48% and 47% in Panc-1 and MIA PaCa-2 cells, respectively. Co-treatment of catalase and EGCG reduced cell growth by 17% and 38% in Panc-1 and MIA PaCa-2 cells (Figure 3d). Of note, this effect was significantly different to that of the EGCG group (*p* < 0.01). Consistently, co-treatment with NAC showed a similar effect as catalase in preventing the reduction in cell growth (Figure 3d). These results indicate that preventing ROS generation partly counteract the effect of EGCG on cell growth inhibition.

The inhibition of Akt activation led to a reduction in mTOR activation in Panc-1 and MIA PaCa-2 cells. In addition, EGCG inhibited upstream events in Akt activation, including IGFR and PI3K phosphorylation (Figure 3e).

### 3.4. EGCG Suppresses Akt Protein Expression by Affecting Protein Transcription, Translation, and Degradation

**Figure 4:**
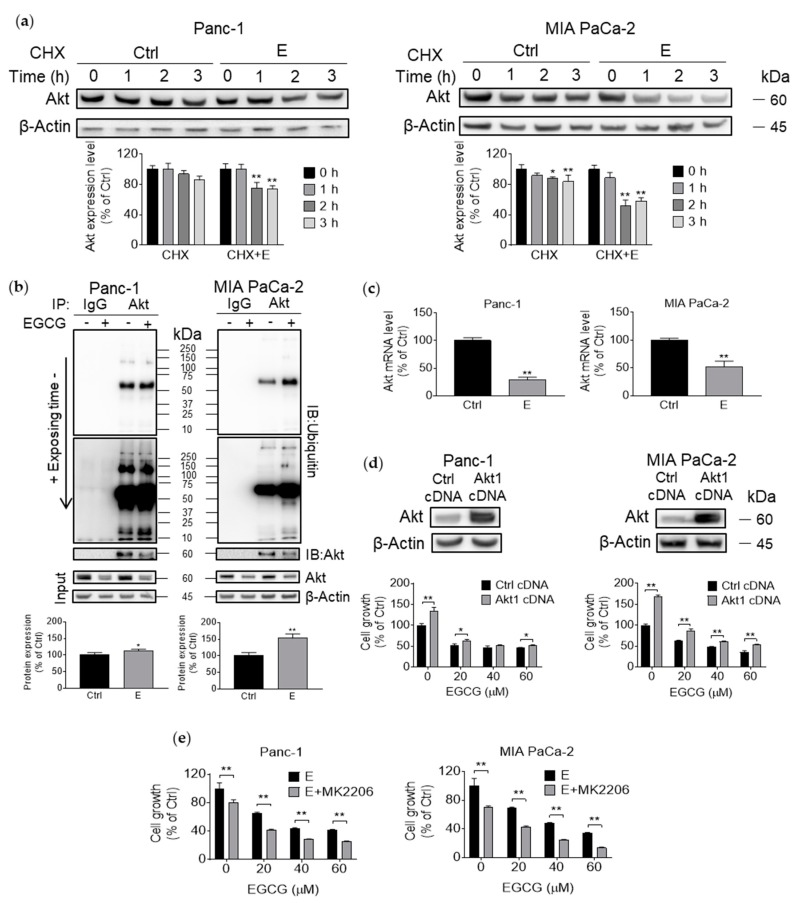
EGCG inhibits Akt protein expression via affecting protein transcription, translation, and degradation. (**a**) Inhibition of protein synthesis with cycloheximide enhanced the effect of EGCG on Akt degradation. Cells were pretreated with or without EGCG for 6 h, then 1–3 h after CHX treatment, a chasing experiment was conducted to measure Akt expression level. Results (mean ± SD) are expressed as a percentage of control. * *p* < 0.05, ** *p* < 0.01 vs. control. (**b**) EGCG enhanced ubiquitination of Akt. Analysis of Akt ubiquitination in Panc-1 and MIA PaCa-2 cells treated with EGCG. After Akt immunoprecipitation, immunoblotting for ubiquitin was performed. Protein input was monitored by direct Western blot. Results are expressed as a percentage of control. * *p* < 0.05, ** *p* < 0.01 vs. control. (**c**) EGCG significantly decreased Akt mRNA levels. Results are expressed as a percentage of control. ** *p* < 0.01 vs. control. (**d**) Akt overexpression ameliorates, in part, the cell growth inhibition by EGCG. Panc-1 and MIA PaCa-2 cells were transfected with a control (cDNA) or Akt-expressing plasmid for 8 h and then treated with EGCG for 72 h. Cell growth was evaluated by the MTT assay. Results are expressed as a percentage of control. * *p* < 0.05, ** *p* < 0.01 vs. control. Top: Akt expression status in whole cell protein lysates following transfection. (**e**) MIA PaCa-2 and Panc-1 cells were treated without or with the Akt specific inhibitor MK2206, EGCG, or both for 72 h. Results are expressed as a percentage of control. * *p* < 0.05, ** *p* < 0.01 vs. control.

Since EGCG reduced both phosphorylation and total Akt levels, we explored whether EGCG affected Akt degradation. Initially, we examined whether Akt is regulated post-translationally by EGCG by pretreating Panc-1 and MIA PaCa-2 cells with EGCG at 40 µM for 6 h and then treating with the protein synthesis inhibitor cycloheximide (CHX) for 1–3 h. Treatment with EGCG accelerated Akt degradation in both cell lines compared with CHX treatment alone, with a stronger effect observed in MIA PaCa-2 cells (Figure 4a).

Given that protein ubiquitination plays an essential role in modulating protein degradation, we immunoprecipitated Akt and assessed whether ubiquitination levels of Akt were affected by EGCG. In MIA PaCa-2 and to a lesser extent in Panc-1 cells, EGCG significantly enhanced Akt ubiquitination (*p* < 0.01 for both; Figure 4b). Finally, we examined the effect of EGCG on transcription levels of Akt by qPCR. EGCG significantly decreased Akt mRNA levels in Panc-1 cells and to a lesser extent in MIA PaCa-2 cells (Figure 4c).

To confirm whether the Akt pathway is a key mechanism of EGCG action, we generated MIA PaCa-2 and Panc-1 cells overexpressing Akt (Akt1 cDNA), with cells transfected with scramble cDNA being our controls (control cDNA). Akt overexpression in the pancreatic cancer cells was confirmed by immunoblotting. Interestingly, the overexpression of Akt abrogated, only partially, the growth inhibitory effect of EGCG. For instance, treatment of Panc-1 and MIA PaCa-2 cells with EGCG at 20 µM for 72 h reduced cell growth by 48% and 38%, respectively. In contrast, overexpression of Akt partially prevented the reduction in cell growth induced by EGCG at 20 µM, with 38% and 14% reductions in cell growth for Panc-1 and MIA PaCa-2 cells, respectively, at 72 h (Figure 4d). On the other hand, treatment with the Akt inhibitor MK2206 for 72 h enhanced the cell growth inhibition effect of EGCG. For example, 40 µM EGCG plus MK2206 decreased cell growth rate to 28% and 24% in Panc-1 and MIA PaCa-2 cells, respectively, while EGCG alone reduced cell growth to 43% and 48%, respectively (*p* < 0.01; Figure 4e).

### 3.5. EGCG Enhances the Growth Inhibitory Effect of Gemcitabine

**Figure 5:**
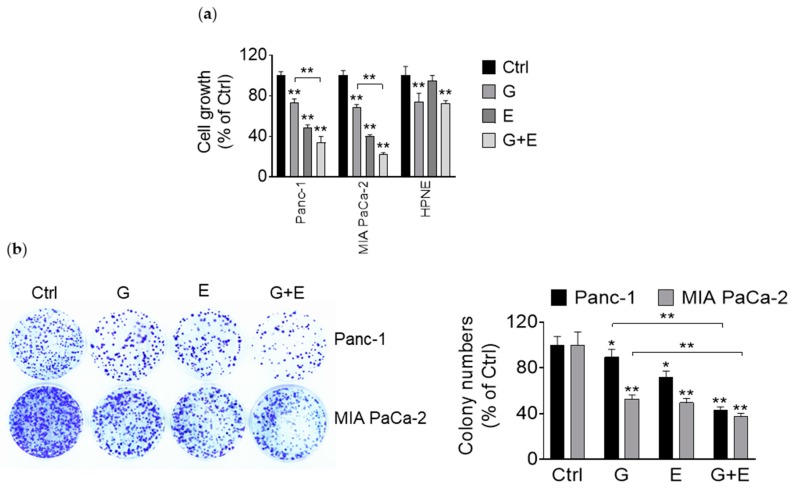
EGCG enhances the cell growth inhibitory effect of gemcitabine in pancreatic cancer cells. (**a**) Cell growth was determined in Panc-1, MIA PaCa-2, and HPNE cells following treatment with EGCG (40 µM), gemcitabine (20 nM), or both for 72 h. Results are expressed as a percentage of control. ** *p* < 0.01 vs. control. (**b**) Panc-1 and MIA PaCa-2 cells were treated with EGCG (E), gemcitabine (G), or both (G + E) for 48 h, and the colony formation capacity was determined. Results are expressed as a percentage of control. * *p* < 0.05, ** *p* < 0.01 vs. control.

Substantial preclinical and clinical evidence supports the notion that a number of combination therapies increase anticancer efficacy while limiting toxicity. Thus, we evaluated in pancreatic cancer cells the efficacy of EGCG in combination with gemcitabine, a current chemotherapeutic used in pancreatic cancer patients. For this purpose, we co-treated Panc-1 and MIA PaCa-2 cells with EGCG alone or in combination with gemcitabine for 72 h and compared the effect of these drugs in the pancreatic cancer cells to that of the HPNE cells. In both Panc-1 and MIA PaCa-2 cell lines, EGCG strongly synergized with gemcitabine (Combination Index = 0.47 and 0.26, respectively; Figure 5a). For example, in Panc-1 and MIA PaCa-2 cells, gemcitabine alone reduced cell growth to 73% and 68%, and once combined with EGCG, the cell growth was further reduced to 33% in Panc-1 and 22% in MIA PaCa-2 cells. Of note, in HPNE cells, gemcitabine alone decreased cell growth rate to 74%, while EGCG failed to further enhance gemcitabine’s effect. Indeed, while in Panc-1 and MIA PaCa-2 cells, EGCG + gemcitabine reduced cell growth by 67% and 78% respectively, cell growth was only reduced by 28% in HPNE cells (Figure 5a).

Given their strongest synergistic effect, we further explored the combination effect of EGCG with gemcitabine. In agreement with the growth inhibitory results, EGCG enhanced the inhibitory effect of gemcitabine on colony formation in Panc-1 and MIA PaCa-2 cells. For example, gemcitabine alone reduced the clonogenic assay by 11% and 47% in Panc-1 and MIA PaCa-2 cells, respectively, and this inhibitory effect was enhanced to 58% and 63% when combined with EGCG (*p* < 0.01 for both; Figure 5b).

### 3.6. EGCG Enhances Gemcitabine’s Inhibition of Pancreatic Cancer Cell Migration and Invasion

**Figure 6:**
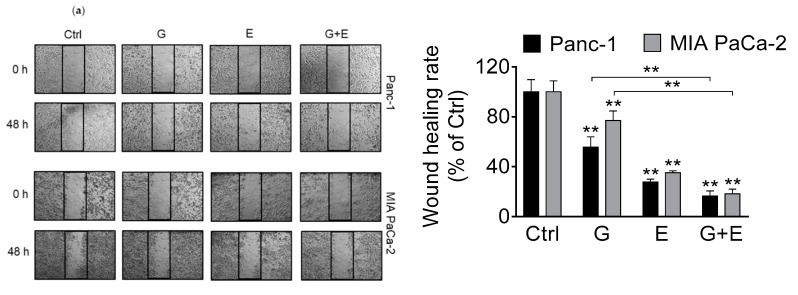

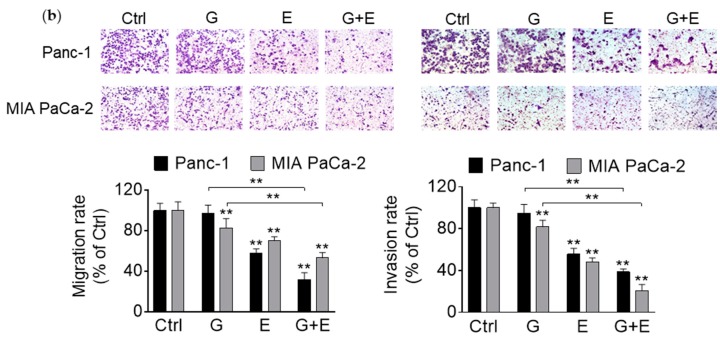
EGCG enhances the inhibitory effect of gemcitabine on cell migration and invasion. (**a**) Panc-1 and MIA PaCa-2 cells were treated with EGCG (E), gemcitabine (G), or both (G + E) for 48 h, and the wound healing rate was determined. Results are expressed as a percentage of control. ** *p* < 0.01 vs. control. (**b**) Migration (left) and invasion (right) rate were further decreased by the combo in Panc-1 and MIA PaCa-2 cells. Representative images are shown (×200). Results are expressed as a percentage of control. ** *p* < 0.01 vs. control.

To determine the effect of EGCG and gemcitabine on pancreatic cancer cell migration and invasion, the wound healing assay was initially performed to evaluate cell motility. Gemcitabine alone reduced the wound healing rate in Panc-1 and MIA PaCa-2 cell lines by 44% and 23%, respectively, compared to vehicle controls (*p* < 0.01 for both). The wound healing rate was further reduced in the EGCG + gemcitabine combination group by 83% in Panc-1 cells (*p* < 0.01) and 82% in MIA PaCa-2 cells (*p* < 0.01 for both; Figure 6a).

Comparable results were observed using the cell migration assay. EGCG plus gemcitabine reduced cell migration by 68% and 47% in Panc-1 and MIA PaCa-2 cells, respectively, whereas gemcitabine alone reduced cell migration by 3% and 17%, respectively (Figure 6b). Of note, these effects were significantly different from those of gemcitabine alone (*p* < 0.01). The invasive rate of both cell lines was further reduced in the EGCG + gemcitabine group by 61% in Panc-1 and 79% in MIA PaCa-2 cells, compared with gemcitabine alone (*p* < 0.01 for both; Figure 6b).

### 3.7. EGCG Sensitizes Gemcitabine on the Inhibition of Akt Pathway and Epithelial–Mesenchymal Transition Markers

**Figure 7:**
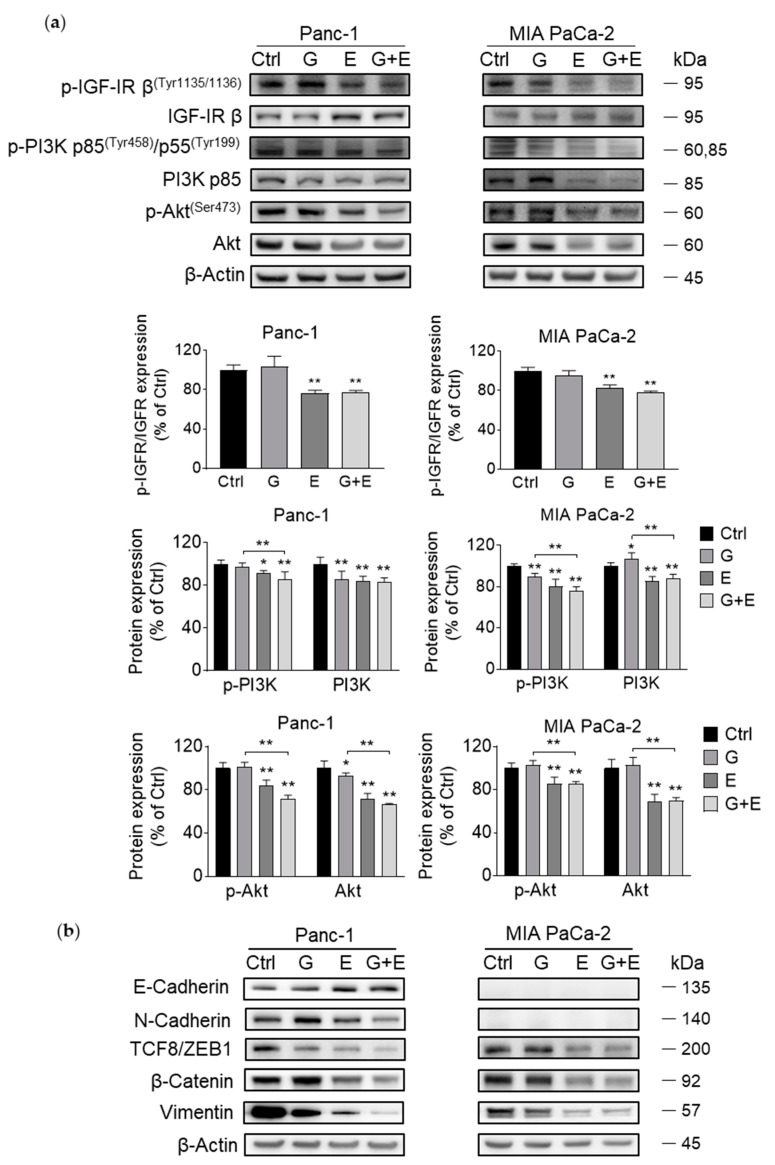

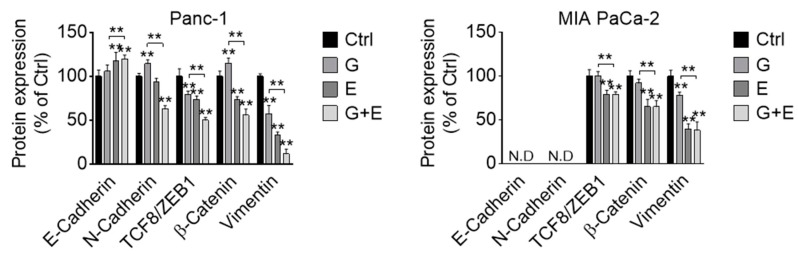
EGCG enhances sensitivity of gemcitabine on Akt and EMT pathway inhibition. (**a**) Co-treated with EGCG (E), the suppression effect of gemcitabine (G) on Akt pathway is enhanced. Immunoblots for phosphorylated/total IGFR, phosphorylated and total PI3K, and phosphorylated and total Akt in whole cell protein extracts from Panc-1 and MIA PaCa-2 cells treated with EGCG (E), gemcitabine (G), or both (G + E) for 12 h. Loading control: β-Actin. Bands were quantified and results are expressed as a percentage of control. * *p* < 0.05, ** *p* < 0.01 vs. control. (**b**) EGCG plus gemcitabine further inhibit EMT. Immunoblots for E-Cadherin, N-Cadherin, TCF8/ZEB1, β-catenin, and Vimentin in whole cell protein extracts from Panc-1 and MIA PaCa-2 cells treated with EGCG (E), gemcitabine (G), or both (G + E), for 48 h. Loading control: β-Actin. Bands were quantified and results are expressed as a percentage of control. ** *p* < 0.01 vs. control.

Given that EGCG affected Akt pathway, we evaluated whether combining EGCG with gemcitabine would lead to any additional inhibitory effect. For this purpose, we tested the level of Akt pathway protein expression in Panc-1 and MIA PaCa-2 cells, though we did not observe any significant additional inhibitory effect of these two drugs on the Akt pathway. But at least, after 12 h, for both cell lines, protein expression in the EGCG + gemcitabine group was similar to that of EGCG alone (Figure 7a).

Moreover, EGCG + gemcitabine showed a stronger inhibitory effect than either agent alone on EMT markers, including increased expression of E-Cadherin, and decreased expression of N-cadherin, TCF8/ZEB1, β-Catenin, and Vimentin (Figure 7b).

## 4. Discussion

A large number of pancreatic cancer patients are diagnosed with metastatic disease and a very poor prognosis. Given that current chemotherapeutics provide limited or no help for patients bearing metastatic pancreatic cancer, new therapeutic strategies are needed, including a combination of drugs that can act additively or synergistically. In this study, we identified EGCG as a potentially safe and effective agent for use with gemcitabine in the blocking of pancreatic cancer migration and invasion partly by inhibiting the Akt pathway and EMT.

Efficacy and safety are critical considerations in the evaluation of anticancer agents [27,28]. Under the conditions tested, EGCG appears to satisfy these two critical requisites: Improved safety and enhanced efficacy. EGCG effectively reduced pancreatic cancer cell growth in vitro and in vivo. In a panel of human pancreatic cancer cells, EGCG reduced pancreatic cancer growth in a concentration and time-dependent manner. Furthermore, based on our in vivo studies, which permitted us to investigate the therapeutic potential of EGCG, the inhibition of tumor growth by EGCG was strong, significantly reducing the rate of growth of the aggressive KPC xenograft tumors by up to 49%. Moreover, our animal studies assessed multiple parameters of toxicity. EGCG was well tolerated, showing essentially no signs of liver and kidney toxicity. Overall, these preclinical models indicate that EGCG may be safe and efficacious in the treatment of pancreatic cancer.

A noteworthy aspect of EGCG, both as a single agent and in combination with gemcitabine, is its selectivity. Anticancer agents should target preferably the tumor and not the normal tissues. EGCG ± gemcitabine exhibits such selectivity. Compared with multiple human pancreatic cancer cell lines, the normal human pancreatic epithelial cells are more resistant to EGCG ± gemcitabine-induced suppression of their growth. Such selectivity, together with lack of apparent toxicity, is a significant advantage of EGCG.

Akt activation is frequent in pancreatic cancer, playing a critical role in promoting tumor growth and metastasis [4,5,6,7]. EGCG inhibited Akt activation through different mechanisms, including block protein transcription, translation, and degradation. Of note, CAT and NAC failed to reverse the effect of EGCG on the Akt pathway, suggesting this action is independent of the pro-oxidant capacity of EGCG. Because Akt signaling plays an important role in pancreatic cancer cell growth, downregulation of this kinase could explain, in part, the reduction in cell growth observed in EGCG-treated tumors. Overall, these findings provide preclinical relevance to the inhibition of Akt by EGCG and suggest that the inhibition of Akt is beneficial in pancreatic cancer, including as a target for combination treatment.

In addition, Akt activation plays an important role in the migration, invasion, metastasis, and chemoresistance of cancer cells by influencing EMT-related gene expression and behavior [29], in which cells acquire migratory and invasive properties and become more resistant to chemotherapy [30]. Moreover, in cells undergoing the EMT process, there will be a “Cadherin Switch”, which refers to the transition from E-Cadherin to N-Cadherin. As an epithelial marker protein, E-Cadherin maintains cell–cell contacts, while N-Cadherin belongs to a mesenchymal marker protein and promotes cell invasion [31]. Vimentin, an intermediate filament modeling the cytoskeleton, is highly expressed in invasive cells [32]. Interestingly, in a mesenchymal state, an increased expression of Vimentin, ZEB1, and Snail, and a decreased expression of E-cadherin is often observed [33]. Of note, ZEB1 can suppress E-Cadherin expression and promote tumor cell dedifferentiation [34]. As a key downstream effector of the Wnt signaling pathway, β-Catenin participates in tumorigenesis and can modulate cell metastasis [35]. In pancreatic cancer cells and mouse xenografts, EGCG alone and in combination with gemcitabine prevented the “Cadherin Switch” as well as downregulated expression of TCF8/ZEB1, Vimentin, and β-Catenin, thus reducing the mesenchymal phenotype.

Current pancreatic cancer chemotherapy includes a combination of drugs to enhance efficacy and minimize toxicity. Gemcitabine, a current drug for metastatic pancreatic cancer, only extends a patient’s life for months; we explored whether EGCG enhances the anticancer efficacy of gemcitabine. EGCG is a promising combination agent for pancreatic cancer therapy, with its enhanced anticancer effect when combined with gemcitabine in vitro and in vivo. Moreover, EGCG enhanced gemcitabine’s suppression of cell migration and invasion in pancreatic cancer cells. Given that Akt activation and EMT promote cells to acquire migratory and invasive properties and become more resistant to chemotherapy [30,36], our findings showing the benefit of combination may have some clinical relevance. Thus, EGCG is a promising combination agent for pancreatic cancer treatment, with its enhanced anti-migration and anti-invasion effect when combined with gemcitabine. In agreement with our findings, EGCG has been shown to increase efficacy of chemotherapeutics and reduce their side effects in various animal tumor models [37].

## 5. Conclusions

EGCG selectively inhibited pancreatic cancer cell growth in vitro and in mouse xenografts. EGCG also effectively inhibited cell migration and invasion, partly by suppressing Akt pathway and EMT. In addition, EGCG showed a strong beneficial effect when combined with gemcitabine. Taken together, our results point to the potential of EGCG to act as an adjuvant therapy for pancreatic cancer.

## Figures and Tables

**Table 1:** Serum levels of multiple biochemical enzymes and markers of liver and kidney function for control, EGCG 10 mg/kg/d, and 20 mg/kg/d at the end of the treatment period. Results are presented as the mean ± SD.

	Alanine Transaminase U/L	Albumin g/dL	Alkaline Phosphatase U/L	Aspartate Transaminase U/L	Blood Urea Nitrogen mg/dL	Creatinine mg/dL	Total Bilirubin mg/dL	Total Protein g/dL
Reference Range	0–403	2.9–4.0	49–172	0–552	15.2–34.7	0.0–0.3	0.0–0.2	4.7–6.1
Ctrl	38.6 ± 12.5	3.1 ± 0.1	72.4 ± 2.0	98.8 ± 17.0	19.8 ± 5.0	0.1 ± 0.1	0.1 ± 0.0	4.6 ± 0.2
E 10mg/kg/d	40.3 ± 17.2	3.3 ± 0.4	118.3 ± 44.5	119.6 ± 58.7	26.0 ± 5.2	0.1 ± 0.0	0.1 ± 0.1	4.9 ± 0.2
E 20mg/kg/d	35.3 ± 18.3	3.6 ± 0.1	132.0 ± 6.7	93.1 ± 11.5	27.0 ± 4.2	0.1 ± 0.0	0.1 ± 0.0	5.0 ± 0.3
